# Preclinical Study of Pain Neuropeptide Expression in Murine Sensory Neurons Induced by Irradiated Osteoclasts in the Context of Stereotactic Body Radiation Therapy

**DOI:** 10.3390/cells14171324

**Published:** 2025-08-27

**Authors:** Sun H. Park, Megan Peters, Caleb Aguayo, Michael K. Farris, Ryan T. Hughes, Joseph Moore, Michael T. Munley, Kaitlyn E. Reno, Jeffrey A. Foster, Jean Gardin, George W. Schaaf, J. Mark Cline, Christopher M. Peters, Jeffrey S. Willey

**Affiliations:** 1 Department of Radiation Oncology, Wake Forest University School of Medicine, Medical Center Boulevard, Winston-Salem, NC 27157, USA; sun.h.park@wakehealth.edu (S.H.P.); meganpetersx08@gmail.com (M.P.); caguayo@wakehealth.edu (C.A.); mfarris@wakehealth.edu (M.K.F.); ryhughes@wakehealth.edu (R.T.H.); jemoore@wakehealth.edu (J.M.); mmunley@wakehealth.edu (M.T.M.); kreno@wakehealth.edu (K.E.R.); gschaaf@wakehealth.edu (G.W.S.); jmcline@wakehealth.edu (J.M.C.); 2 Department of Orthopaedic Surgery, Wake Forest University School of Medicine, Winston-Salem, NC 27157, USA; jeafoste@wakehealth.edu; 3 Department of Pathology, Section on Comparative Medicine, Wake Forest University School of Medicine, Winston-Salem, NC 27157, USA; jgardin@wakehealth.edu; 4 Department of Anesthesiology, Wake Forest University School of Medicine, Winston-Salem, NC 27157, USA; chrpeter@wakehealth.edu

**Keywords:** stereotactic body radiation therapy, chest wall pain, osteoclasts, sensory neurons, bisphosphonates, neuropeptides

## Abstract

Stereotactic body radiation therapy (SBRT) for lung tumors near the chest wall often causes significant chest wall pain (CWP), negatively impacting patients’ quality of life. The mechanisms behind SBRT-induced CWP remain unclear and may involve multiple factors. We investigated crosstalk between radiation-activated osteoclasts and sensory neurons, focusing on osteoclast-derived factors in CWP. Using murine pre-osteoclast cell line Raw264.7, we induced differentiation with Receptor Activator of Nuclear Factor kappa-beta Ligand (RANKL), followed by 10 Gy gamma-irradiation. Conditioned media (C.M) from irradiated osteoclasts was used to treat sensory neuronal cultures from mouse dorsal root ganglia. Neuronal cultures were also exposed to 10 Gy radiation, with and without osteoclast co-culture. Osteoclast markers and pain-associated neuropeptides were analyzed using RT-qPCR and histochemical staining. Osteoclasts differentiation and activity were inhibited using osteoprotegerin (OPG) and risedronate. High-dose radiation significantly increased the size of tartrate-resistant-acid-phosphatase (TRAP)-positive osteoclasts (1.36-fold) and activity biomarkers (*Ctsk*, 1.35-fold, *Mmp9*, 1.76-fold). Neurons treated with C.M from irradiated osteoclasts showed ~1.5-fold increase in *Calca* (calcitonin gene-related peptide) and *Tac1* (substance P) expression, which was mitigated by osteoclast inhibitors. These findings suggest that radiation enhances osteoclast activity and promotes pain signaling. Osteoclast inhibitors may represent a therapeutic strategy to reduce CWP and improve quality of life.

## 1. Introduction

Stereotactic body radiation therapy (SBRT) is a standard treatment for patients with lung tumors who decline, or are ineligible for, surgical resection [1]. For peripheral lung tumors near the chest wall, SBRT often delivers high radiation doses to nearby ribs or vertebrae, predisposing patients to chest wall pain (CWP), radiation-induced rib fractures (RIRF), or even vertebral fractures [2,3,4].

CWP occurs in approximately one third of patients when greater than 30 cm^3^ of chest wall receives 30 Gy or more [4]. In clinical practice, it is common to prioritize comprehensive target coverage over sparing of non-critical organs at risk, such as the chest wall and ribs, to maintain optimal tumor control outcomes [5,6]. However, concerns over severe chest wall toxicity may lead some clinicians to lower the radiation dose or use protracted fractionation schemes, which can compromise survival outcomes [5].

CWP usually manifests between 6 and 9 months post-treatment and may occur with or without evidence of rib fractures [4,7,8,9,10]. The incidence of RIRF within two years after SBRT is as high as 40% [11], often causing sharp pain that often persists until fractures heal. SBRT-associated RIRFs demonstrate delayed healing compared to traumatic fractures; in one study, only 47% of RIRF healed by a median 49-month follow-up [2].

CWP without radiologic fracture may present as acute, focal sharp pain lasting days to weeks, or as chronic, dull pain for years, and can significantly impair patients’ quality of life [4,11,12]. Severe pain may lead to compromised ventilation, decreased oxygen saturation, and increased risks of atelectasis and infection [13]. Pain management can be challenging and may involve non-steroidal anti-inflammatory drugs, corticosteroids, gabapentin, narcotics, and/or percutaneous nerve blocks [11,14]. Importantly, these treatments only offer symptomatic relief without addressing the root cause of the CWP. This underscores the urgent need for targeted strategies that mitigate SBRT-related CWP/RIRF without compromising cancer control.

The mechanisms underlying SBRT-induced CWP are not fully understood, but may include localized inflammation, dysregulated bone turnover (via differential effects on osteoclasts and osteoblasts), fibrosis, nerve injury, or a complex mix of all of these mechanisms [11,15,16,17]. Our prior research indicates that low-dose radiation increases osteoclast number and activity, resulting in increased bone degradation [18,19,20,21,22,23], and that preemptive administration of bisphosphonates can counter radiation-induced bone loss in animal models [24]. This suggests that early interventions targeting osteoclast activation might help prevent CWP, especially those cases associated with rib fractures [25,26,27].

However, this does not account for CWP cases that occur without fractures. Active osteoclasts may release factors including exosomes and acidic byproducts that promote sensory nerve growth and pain similar to what has been observed in osteoarthritis and cancer-induced bone pain models [28,29,30]. Importantly, it is unknown whether radiation-activated osteoclasts contribute to neuronal sensitization or pain signaling following SBRT. This represents a critical knowledge gap, as no prior studies have directly investigated how osteoclast-derived signals affect sensory neurons in the setting of radiation exposure and CWP.

We hypothesized that high-dose radiation enhances osteoclast activity, leading to the release of secretory factors that activate pain signaling in sensory neurons. To test this, we used an in vitro model combining radiation-activated murine osteoclasts and primary sensory neuronal cultures to evaluate osteoclast–neuron interactions and determine their effects on pain-associated neuropeptide expression. We also examined whether osteoclast inhibitors (osteoprotegerin [OPG] and risedronate) could mitigate these effects.

## 2. Materials and Methods

### 2.1. Animals

All animal work was performed as approved by the Wake Forest University School of Medicine (WFUSM) Institutional Animal Care and Use Committee (IACUC). WFUSM is accredited by the Association for Assessment and Accreditation of Laboratory Animal Care, International, and is compliant with all applicable animal welfare laws and regulations (Office for Protection from Research Risks Assurance #A-3391-01). The study was carried out in compliance with the Animal Research: Reporting of In Vivo Experiments (ARRIVE) guidelines and in accordance with relevant guidelines and regulations. Tissues were obtained from animals solely for the purpose of in vitro studies, and the study adhered to all relevant ethical guidelines and institutional regulations governing the use of animals in research.

### 2.2. Radiation Treatment

All cell irradiations were performed using a Cs-137 γ irradiator (Model [68A], J.L. Shepherd & Associates, San Fernado, CA, USA). Cell culture plates (e.g., 24-well, 96-well) were placed in a custom template designed to fit the irradiator chamber. Plates were positioned centrally within the chamber to ensure uniform exposure. After closing the cavity door, a total dose of 10 Gy (equivalent to 1000 Rad) was delivered over 3.67 min, corresponding to a dose rate of approximately 273 rad/min. Following exposure, cells were immediately returned to standard culture conditions in a humidified incubator at 37 °C with 5% CO_2_.

### 2.3. Sensory Neuronal Cultures from Mice Dorsal Root Ganglia (DRG)

Mice were purchased from The Jackson Laboratory (Bar Harbor, ME, USA). Mice were euthanized with CO_2_ followed by cervical dislocation, in accordance with IACUC guidelines. For each mouse DRG culture experiment, 3–4 mice were euthanized depending on the number of treatment groups included in the study. Primary DRG neuronal culture was prepared following the previously published protocol with modifications [31]. Thoracic DRG (T1–T13) of male C57BL/6 mice (8–12 weeks old) were dissected followed by enzymatic digestions: (1) tissues were incubated in 3 mL of papain solution containing 30 U/mL papain (Worthington Biochemical Corp., Lakewood, NJ, USA), 0.1% saturated NaHCO_3_ solution (Sigma-Aldrich, St. Louis, MO, USA), and 0.3 mg/mL L-Cys (Sigma-Aldrich) for 30 min at 37 °C with 5% CO_2_; and they were (2) incubated in 3 mL of collagenase type II (CLS2)/dispase type II (Dispase II) solution containing 4 mg/mL CLS2 (Worthington Biochemical Corp.) and 4.7 mg/mL dispase (Sigma-Aldrich) for 30 min at 37 °C with 5% CO_2_. DRG tissues were triturated and filtered through a stainless mesh sieve (40 µm, Thermo Fisher Scientific, Waltham, MA, USA) to obtain single-cell suspensions. Cells were incubated with myelin beads (Miltenyi Biotec, Gaithersburg, MD, USA) in bovine serum albumin (BSA) buffer for 10 min at 4 °C following the company’s protocol. The bead/cell suspension went through the magnetic column to remove myelin debris, and the eluted cell suspension was washed and counted. A total of 2 × 10^3^–5 × 10^3^ cells of DRGs in 30 µL of warm neuronal growth (NG) medium [Neurobasal-A, 1% N_2_, 2% B-27, 2 mM L-glutamine, 1% penicillin-streptomycin (All purchased from Thermo Fisher Scientific, Gibco, Waltham, MA, USA), and 0.4% glucose (Sigma-Aldrich)] were seeded onto the center of 12 mm round coverslips (MatTek Corp., Ashland, MA, USA), pre-coated with Poly-D-lysine (50 µg/mL, overnight at 4 °C, Thermo Fisher Scientific, Waltham, MA, USA, Corning, Corning, NY, USA) and laminin (20 µg/mL, >1 h at room temperature (RT), Thermo Fisher Scientific, Corning), in 24-well plate. After 1–2 h, 1 mL of warm NG medium was gently added to the sides of wells and the cells were maintained at 37 °C with 5% CO_2_. Once a total of 48 h had passed after cells were seeded, cells were treated with radiation using Cs137 γ-rays and/or conditioned media of radiation activated osteoclasts for further studies. For co-cultures, 2 × 10^3^ cells/60 µL growth media were plated on µ-Slide 2 Well Co-Culture plates (81806, ibidi USA, Inc., Fitchburg, WI, USA).

### 2.4. Sensory Neuronal Cultures from Non-Human Primate (NHP) DRGs

DRGs were harvested from rhesus macaques (Macaca mulatta), non-human primates that were enrolled as part of the National Institutes of Health and National Institute of Allergy and Infectious Diseases-supported Radiation Late Effects Cohort at WFUSM) [32]. NHP primary neuronal culture was prepared similarly as described in the murine primary neuronal culture following the published study [31]. Briefly, thoracic DRGs were collected from two deceased male rhesus macaques, one 22-year-old with no radiation history and one 13-year-old exposed 9 years prior to a single fraction of 8 Gy total body irradiation. Both animals were captive-bred and imported from China prior to adoption by WFUSM, and both were nominally of Chinese genetic origin. The DRGs were dissected, directly placed in a 15 mL conical tube containing 14 mL of ice-cold Hanks’ Balanced Salt Solution (HBSS) without Mg^++^/Ca^++^ (Fisher Scientific, Hanover Park, IL, USA), and transferred to the laboratory for further procedures. Isolated DRGs were cleaned up and digested through a series of enzymatic treatments using papain and collagenase type II (CLS2)/Dispase II solutions. Afterwards, single-cell-suspended DRG neurons were filtered and further purified by centrifugation in 3.5% (*w*/*v*) BSA solution. Cells were then seeded onto the center of 12 mm round coverslips, and pre-coated with Poly-D-lysine and laminin, in 24-well plate. After 72 h, cells were treated with radiation using Cs137 γ-rays at a total dose of 10 Gy.

### 2.5. Culturing and Differentiation of Osteoclast

The murine pre-osteoclast cell line, Raw264.7, from American Type Culture Collection (ATCC) was cultured in alpha minimum essential medium (αMEM) supplemented with 10% fetal bovine serum (FBS), 1% penicillin-streptomycin, and 1% glutamine. Cells were passaged every 2–3 days when cells were close to 80% confluent. Cells passaged less than 15 times were used for all the experiments. To differentiate Raw264.7 cells, 3 × 10^3^ cells were plated in 96-well plates or 3.2 × 10^4^ cells in 24-well plates in growth media containing 35 ng/mL of murine recombinant receptor activator of nuclear factor kappa-beta ligand (RANKL) (462-TEC-010, R&D systems Inc., Minneapolis, MN, USA). Differentiated osteoclasts (identified by bright field microscopy based on their larger size, spread-out morphology, and multinucleation compared to undifferentiated controls) were detected on day 3 post addition of RANKL. For co-cultures, 2 × 10^3^ cells/60 µL growth media were plated on µ-Slide 2 Well Co-Culture plates (81806, ibidi USA, Inc., Fitchburg, WI, USA) in the presence of 35 ng/mL RANKL. To inhibit osteoclast differentiation and activity, osteoclast cultures were treated with recombinant murine OPG (459-MO-100, R&D systems Inc.) or a bisphosphonate (risedronate, S1874, Selleck Chemicals LLC, Houston, TX, USA) a day before radiation treatment.

### 2.6. Tartrate-Resistant Acid Phosphatase (TRAP) Staining and Image Analysis

Cells were washed with 1x PBS solution and fixed with a 10% glutaraldehyde solution (Sigma-Aldrich) for 15 min at 37 °C. The cells were then washed twice with pre-warmed 1× PBS and stained with TRAP staining solution containing 0.3 mg of Fast Red Violet LB Salt per mL of TRAP buffer [50 mL of 0.1 M acetate solution, 10 mL of 0.3 M sodium tartrate, 1 mL of 10 mg/mL Napthol solution, 100 μL of Triton X-100, and 38.9 mL of double-distilled water (ddH_2_O), pH 5] for 10 min at 37 °C. After removing the TRAP staining solution, the cells were washed with 1× PBS and air-dried. Images were acquired on an Olympus IX70 inverted microscope with a DP80 camera at 10× magnification. Four images were taken for each well. The images were captured and processed with CellSens software (version 2.3, Olympus Corporation, Waltham, MA, USA). Using Nikon image analysis software version 5.02.01, TRAP-positive multinucleated mature osteoclasts (containing three or more nuclei) were masked, and the total area masked was calculated and normalized to the total number of osteoclasts within each image to demonstrate changes in osteoclast maturation and activity level. Image software used was the following: The Nikon Elements Basic Research version 5.02.01. 64bit (https://www.microscope.healthcare.nikon.com/products/software/nis-elements, accessed on 20 August 2025).

### 2.7. Resorption Pit Assay for Osteoclast

Raw264.7 cells (3 × 10^3^) were seeded on each 96-well OsteoAssay plates (Fisher Scientific, Corning, NY, USA) in 100 µL of growth media containing RANKL (35 ng/mL). Three days after differentiation, cells were exposed to radiation. 48 h after radiation treatment, cells were removed with 10% bleach solution for 5 min. Each well was washed with distilled water and stained with 1% toluidine blue solution for 10 min at RT, washed with distilled water, and air dried. Images of individual pits or multiple pit clusters were acquired on an Olympus IX70 inverted microscope with a DP80 camera (Olympus Corporation, Hachioji, Tokyo, Japan) at 10× magnification. Image software used was the following: The Olympus cellSens Standard version 2.3 (https://www.olympus-lifescience.com/en/software/cellsens/, accessed on 1 January 2024).

### 2.8. Conditioned Media (C.M) Generation

To collect the C.M of radiation activated mature osteoclasts, 3.2 × 10^4^ of RAW264.7 cells were seeded on 24-well plates in the presence of 35 ng/mL RANKL. After 3 days of differentiation, cells were re-fed with fresh growth media with RANKL (35 ng/mL) and treated with 10 Gy radiation using Cs137 γ-rays. The control cells were cultured in the same manner but were not treated with radiation. The cells were then maintained in a 37 °C incubator with 5% CO_2_. After 48 h, media was collected and centrifuged at full speed for 2 min to remove debris, and supernatant was transferred to new tubes.

### 2.9. RT-qPCR

Cells were lysed in 350 µL of RLT+ beta-mercaptoethanol (β-ME) buffer. RNA was extracted using the Universal RNeasy plus kit (Qiagen, Germantown, MD, USA), and cDNA was generated using Ezdnase enzyme (11766051, Thermo Fisher Scientific) and SuperScript^TM^ IV VILO mater mix (11-756-050, Thermo Fisher Scientific) following the company’s protocol. Quantitative reverse transcription polymerase chain reaction (RT-qPCR) was performed using TaqMan Universal PCR master mix (Applied Biosystems, Foster City, CA, USA) and TaqMan gene expression assays on the Applied Biosystem PCR instrument. TaqMan gene expression assays used were as follows: NGF/*Ngf* (Mm00443039_m1) and CGRP/*Calca* (Mm00801463_g1) and Substance P/*Tac1* (Mm01166996_m1) were used for pain associated biomarkers. CTSK/*Ctsk* (Mm00484039_m1), RANK/*Tnfrsf11a* (Mm00437132_m1), MMP9/*Mmp9* (Mm00442991_m1), and DcStamp/*Dcstamp* (Mm04209236_m1) were used for osteoclast cell markers. Data is presented using the delta-delta Ct method, with GAPDH/*Gapdh* (Mm99999915_g1) or β-actin/*Actb* (Mm02619580_g1) used as the reference gene.

### 2.10. Immunofluorescence Staining

Fixed cells were blocked with immunofluorescence (IF) buffer [1× D-PBS supplemented with 5% Normal Donkey serum (Jackson ImmunoResearch, West Grove, PA, USA) and 0.03% Triton X-100 (Sigma-Aldrich)] for 1 h at RT and incubated with primary antibodies overnight at 4 °C in IF buffer. Primary antibodies used were as follows: mouse anti-β III tubulin antibody (1:1000, Biolegend, San Diego, CA, USA, cat #: 801201); chicken anti-200 kDa neurofilament (NF200) antibody (1:3000, Neuromics, Cambridge, MA, USA, cat #: CH22104); and rabbit anti-CGRP antibody (1:5000, Sigma-Aldrich, cat #: C8198). Afterwards, the cells were incubated with secondary antibodies for 2 h at RT in IF buffer. Secondary antibodies used were as follows: anti-rabbit cyanine 3 (CY3) (1:700, Jackson ImmunoResearch, cat #: 711-165-152); anti-mouse CY2 (1:600, Jackson ImmunoResearch, cat #: 715-225-150); and anti-chicken CY5 (1:500, Jackson ImmunoResearch, cat #: 703-225-155). After washing 5 times with 1× D-PBS, the cells were mounted with ProLong Gold antifade mountant with 4′,6-diamidino-2-phenylindole (DAPI) (Thermo Fisher Scientific).

### 2.11. Neurite Length Analysis

For each group, a total of 3 images (1 large image/coverslip for triplicates) were taken using a Nikon Eclipse Ni fluorescent microscope system (Nikon, Tokyo, Japan) to capture the total neuronal culture. Images were saved as nd2 files for further analysis using Visiopharm following the previously published study (Hørsholm, Denmark). An algorithm (called an “APP”) created in the Visiopharm software was modified to mask total soma and neurites of the neurons in images taken for each experiment. Batch analysis was run for each study using the APP to generate the total nerve length and soma count. The final data were presented as normalized neurite length, calculated by dividing the total neurite length by the total number of somas. Image software used was as follows: Visiopharm version 2023.01.2 (https://visiopharm.com/visiopharm-digital-image-analysis-software-features/, accessed on 1 January 2024). 

### 2.12. Statistical Analysis

Numerical data are expressed as means ± the standard error of the mean (SEM). Statistical analysis was performed by unpaired two-tailed Student’s *t* test or One-way ANOVA with Tukey’s post hoc test, using the GraphPad Prism (Version 10.4.0) statistical program (GraphPad Software, San Diego, CA, USA) with significance at *p* ≤ 0.05.

## 3. Results

### 3.1. Impact of High-Dose Radiation on Osteoclast Maturation and Activity

**Figure 1:**
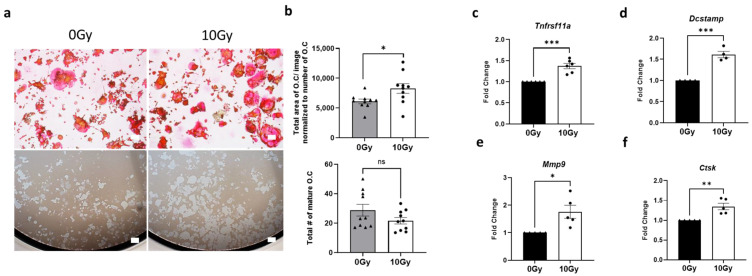
Maturation and activation of osteoclasts following high-dose radiation exposure. (**a**) Representative images showing TRAP staining (top panel) and resorption pit data (bottom panel) for Raw264.7-derived matured osteoclasts (induced by 35 ng/mL RANKL) 48 h after exposure to either no radiation (0 Gy) or a single fraction of 10 Gy Cs-137 γ-radiation. Scale bar = 200 µm. (**b**) Quantification of the total area (top panel) and number (bottom panel) of TRAP-positive mature osteoclasts exposed to no radiation therapy (0 Gy) or radiation therapy (10 Gy). The experiment was performed two times with ten biological replicates. Each data point represents the area from each image analyzed. (**c**–**f**) RT-qPCR analysis of matured osteoclasts exposed to either no radiation (0 Gy) or a single fraction of 10 Gy Cs-137 γ-radiation, illustrating fold change in the expression of genes associated with osteoclast differentiation and maturation (*Tnfrsf11a* and *Dcstamp*), as well as activity (*Mmp9*, *Ctsk*). The figure represents data combined from 5 to 6 independent experiments. Unpaired Student’s *t*-test was used for all statistical analyses. Levels of statistical significance are indicated as * *p* ≤ 0.05, ** *p* ≤ 0.005, and *** *p* ≤ 0.0001. ns: not significant.

To assess the impact of high-dose radiation on osteoclast activity, pre-osteoclast RAW264.7 cells were cultured with 35 ng/mL of RANKL. Following three days of culture, cells were subjected to radiation as described in the methods. We selected 10 Gy based on prior studies showing rapid bone loss in SBRT-relevant models, including non-human primates. This dose also reflects clinical practice, as it is within the typical range used in SBRT regimens for peripheral lung tumors near the chest wall. Forty-eight h post-irradiation, cells were stained with TRAP staining solution to identify mature osteoclasts. We observed a significant increase (fold change = 1.36, *p* = 0.0269) in the size of TRAP-positive osteoclasts in irradiated cells compared to controls (Figure 1a,b). Furthermore, when irradiated cells were plated on bone biomimetic synthetic surfaces, a notable augmentation in resorption pit size was evident compared to untreated control cells (Figure 1a). Complementing these morphological observations, RT-qPCR analysis revealed a robust upregulation in the expression of osteoclast biomarkers associated with differentiation (*Tnfrsf11a:* fold change = 1.37, *p* = 0.0002), fusion (*Dcstamp:* fold change = 1.61, *p* = 0.0002), and resorption activity (*Ctsk:* fold change = 1.35, *p* = 0.003 and *Mmp9:* fold change = 1.76, *p* = 0.0127) in irradiated cells compared to control cells (Figure 1c–f). Notably, expression of *Tnfrsf11a* (RANK), an osteoclast differentiation marker, and *Mmp9* was not significantly elevated at 24 h post-radiation. However, by 48 h, we observed a consistent increase in the expression of both osteoclast differentiation (*Tnfrsf11a*) and activity (*Mmp9 and Ctsk*) markers, indicating enhanced osteoclast maturation and resorptive function at this later time point, as shown in Figure 1a,b. Therefore, all subsequent experiments were performed at 48 h time point.

### 3.2. Impact of Radiation-Activated Osteoclast-Derived Secretory Factors on Pain-Associated Neuropeptides in Sensory Neurons

Building upon previous studies indicating the involvement of secretory factors released from activated osteoclasts in modulating sensory neurons under various pathological conditions, we sought to investigate the impact of radiation-activated osteoclast-derived secretory factors on both the growth of sensory neurons and the expression levels of pain-related factors. These structural and molecular changes are closely associated with the neuronal sensitization processes underlying pain [33,34].

Conditioned media from osteoclasts exposed to 10 Gy radiation were collected and utilized to treat primary sensory neuronal cultures derived from DRG. Control cultures received C.M from non-irradiated osteoclasts. Subsequently, DRG neuronal cultures were fixed and stained with neuronal markers. β-III tubulin served as a pan-neuronal marker, while CGRP was used to identify peptidergic unmyelinated C-fibers and thinly myelinated Aδ-fibers, and neurofilament protein (NF200) identified myelinated A-fibers. These markers allowed for the assessment of changes in neuronal morphology and subtype-specific populations. Also, we specifically measured the levels of pain associated molecules, including neuropeptides (CGRP and Substance P) and nerve growth factors (NGF) as they are well-documented to play crucial roles in mediating pain transmission and modulation. Elevated expression of these neuropeptides has been linked to heightened pain sensitivity in various pain states [33,34], hence their assessment served as a potential indicator of pain-associated responses elicited by radiation-activated osteoclast-derived secretory factors.

**Figure 2:**
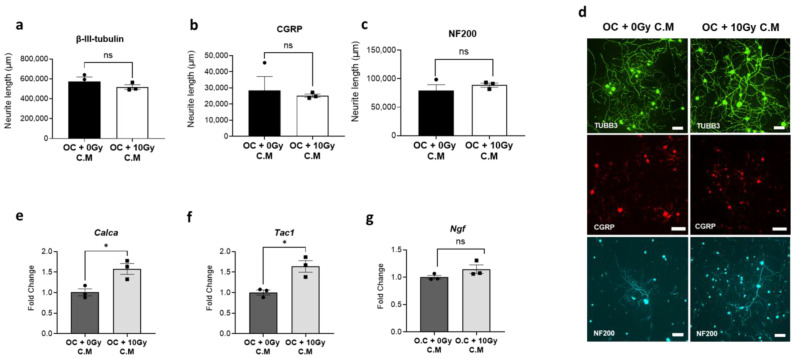
Impact of radiation-activated osteoclasts on murine sensory neuronal cultures. Immunofluorescence analysis of mouse DRG-derived primary sensory neuronal cultures exposed to C.M from Raw264.7-derived matured osteoclasts (induced by 35 ng/mL RANKL) treated with either no radiation (0 Gy) or 10 Gy Cs-137 γ-radiation. After 48 h, cells were stained with antibodies against neuronal markers, (**a**) β-III tubulin, (**b**) CGRP, and (**c**) NF200, followed by microscopic image analysis. (**d**) Representative images of sensory neuronal cultures stained with these neuronal markers, β-III tubulin (TUBB3), CGRP, and NF200. The experiment was performed three times, each time with three biological replicates. The bar graph displays representative results from one independent experiment. Scale bar = 100 μm. (**e**–**g**) mRNA expression levels of pain-associated molecules (CGRP/*Calca*, SP/*Tac1*, and NGF/*Ngf*) in neuronal cultures exposed to C.M from radiation-activated matured osteoclasts (induced by 35 ng/mL RANKL). The experiment was performed three times, each time with three biological replicates. The bar graph displays representative results from one independent experiment. Unpaired Student’s *t*-test was used for all statistical analyses. Levels of statistical significance are indicated as * *p* ≤ 0.05. ns: not significant.

Our data showed that C.M derived from radiation-activated osteoclasts did not exert a significant impact on the growth of sensory neurons. Total neurite length, measured by β-III tubulin staining, was not changed. In addition, when neurite outgrowth was analyzed specifically in CGRP-positive and NF200-positive neuronal subpopulations, no significant differences were observed compared to controls (Figure 2a–d). However, the same C.M from radiation-activated osteoclasts notably increased the mRNA expression of *Calca* (fold change = 1.58, *p* = 0.0027) and *Tac1* (fold change = 1.64, *p* = 0.0143) (Figure 2e,f). No significant change in the expression level of *Ngf* was observed (Figure 2g), which is consistent with the lack of change in neurite length.

### 3.3. Modulation of Radiation-Induced Osteoclast Activation and Neuronal Responses by OPG and Bisphosphonate

**Figure 3:**
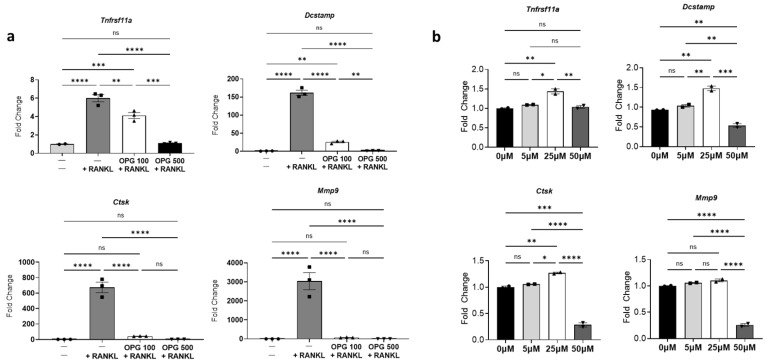
Optimization of OPG and risedronate concentrations on RANKL-induced osteoclast differentiation and activity. Raw264.7-derived matured osteoclasts (induced by 35 ng/mL RANKL) were treated with different concentrations of osteoclast inhibitors, OPG, and bisphosphonate (risedronate), for 48 h and lysed for RT-qPCR analysis. (**a**) mRNA expression levels of mouse osteoclast-associated biomarkers influenced by varying concentrations of OPG (0, 100, 500 µM). The experiment was performed one time with three biological replicates. The bar graph displays representative results from one independent experiment. (**b**) mRNA expression levels influenced by different concentrations of risedronate (0, 5, 25, 50 µM). The experiment was performed one time with two biological replicates. The bar graph displays representative results from one independent experiment. One-way ANOVA with Tukey’s post hoc test was used for all statistical analyses. * *p* ≤ 0.05, ** *p* ≤ 0.005, *** *p* ≤ 0.0005, and **** *p* ≤ 0.0001. ns: not significant.

**Figure 4:**
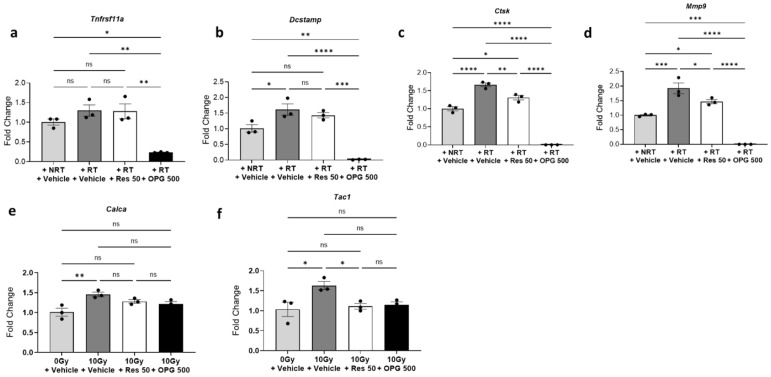
Reduction in neuropeptide expression by osteoclast inhibitors in radiation-activated osteoclast-derived C.M (**a**–**d**) RT-qPCR analysis of Raw264.7-derived matured osteoclasts (induced by 35 ng/mL RANKL), activated by 10 Gy Cs-137 γ-radiation and pre-treated with either OPG (500 µg/mL) or risedronate (50 µM). The experiment was performed two times with three biological replicates. The bar graph displays representative results from one independent experiment. (**e**,**f**) mRNA expression levels of CGRP/*Calca* and SP/*Tac1* in primary murine DRG-derived neuronal cultures exposed to C.M from matured osteoclasts treated, with either no radiation (0 Gy) or 10 Gy Cs-137 γ-radiation, and pre-treated with OPG or risedronate. The experiment with OPG and risedronate was performed one time with three biological replicates. The bar graph displays representative results from one independent experiment. One-way ANOVA with Tukey’s post hoc test was used for all statistical analyses. Levels of statistical significance are indicated as * *p* ≤ 0.05, ** *p* ≤ 0.005, *** *p* ≤ 0.0005, and **** *p* ≤ 0.0001. ns: not significant.

To determine if activated osteoclasts upregulate neuropeptides in neuronal cultures and test the potential of OPG and risedronate as modulators of radiation-induced osteoclast activation and subsequent neuronal responses, osteoclasts were pre-treated with either OPG or risedronate before radiation exposure. First, the concentrations of OPG and risedronate required to inhibit differentiation and activity of osteoclast induced by RANKL were determined. Pre-treatment with 500 µM OPG effectively prevented both RANKL-mediated osteoclast differentiation and activity, as evidenced by decreased expression of differentiation and activity markers (Figure 3a). Conversely, risedronate, up to a concentration of 50 µM, did not impact osteoclast differentiation but effectively inhibited RANKL-mediated osteoclast activity, indicated by decreased expression of *Mmp9* and *Ctsk* (Figure 3b). Subsequently, the effects of OPG and risedronate on radiation-induced osteoclast differentiation, maturation, and activity were investigated. Pre-treatment with OPG (500 µM) significantly attenuated the upregulation of differentiation and activity markers observed in irradiated osteoclasts (Figure 4a–d). Similarly, pre-treatment with risedronate decreased the radiation-induced expression of osteoclast activity markers (*Ctsk, Mmp9*) without significantly impacting differentiation/maturation markers (*Tnfrsf11a* and *Dcstamp*) (Figure 4a–d).

Of particular interest, when neuronal cultures were exposed to C.M derived from irradiated osteoclasts pre-treated with risedronate or OPG, a marginal reduction in the expression of *Calca* (~1.2-fold reduction), and a notable reduction in the expression of *Tac1* (~1.48-fold reduction) was observed, resembling the levels seen in neuronal cultures treated with control C.M (Figure 4e,f).

### 3.4. Effect of Radiation Exposure on Osteoclast-Sensory Neuron Co-Cultures

To replicate the clinical scenario of simultaneous radiation exposure of ribs and intercostal nerves during SBRT to a peripheral lung tumor, osteoclasts and sensory neurons were co-cultured using an indirect non-contact system to focus on paracrine interactions. Both cell types were irradiated simultaneously (10 Gy), followed by media exchange to investigate the effects of secretory factors released by radiation-activated osteoclasts on sensory neurons. This design was intentionally selected to exclude direct cell–cell contact, which could introduce additional confounding variables unrelated to soluble factor signaling. After 48 h of media sharing, the expression levels of neuropeptides in neuronal cultures were assessed.

**Figure 5:**
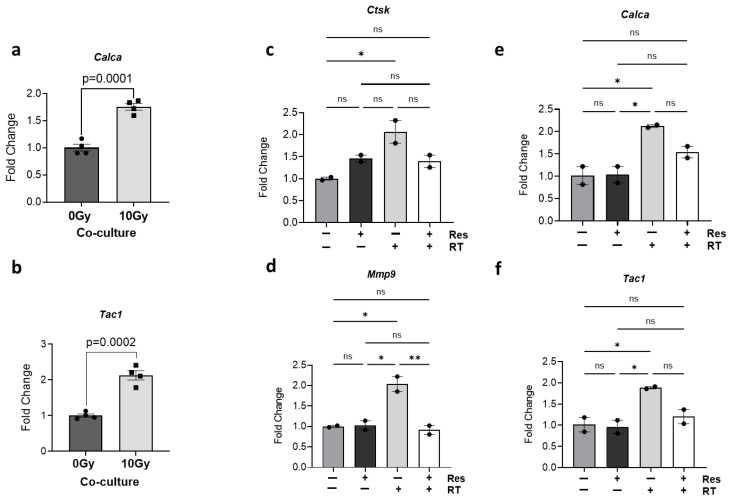
Inhibition of neuropeptide expression in murine sensory neurons co-cultured with osteoclasts by risedronate. (**a**,**b**) Gene expression levels of CGRP/*Calca* and SP/*Tac1* in murine sensory neurons co-cultured with matured osteoclasts (induced by 35 ng/mL RANKL) following simultaneous radiation treatment (10 Gy Cs-137 γ-radiation). The experiment was performed two times with four biological replicates. The bar graph displays representative results from one independent experiment. Unpaired Student’s *t*-test was used for statistical analysis. Gene expression levels of (**c**,**d**) osteoclast activity markers *Mmp9* and *Ctsk* in osteoclasts, and (**e**,**f**) pain-associated neuropeptides, CGRP/*Calca* and SP/*Tac1* in neurons from osteoclast–neuron co-cultures following simultaneous radiation treatment and pre-treatment with risedronate (50 µM). The experiment with risedronate was performed one time with two biological replicates. The bar graph displays representative results from one independent experiment. One-way ANOVA with Tukey’s post hoc test was used for statistical analysis. Levels of statistical significance are indicated as * *p* ≤ 0.05 and ** *p* ≤ 0.005. ns: not significant.

Consistent with previous results utilizing C.M (Figure 2e,f), a significant increase in the gene expression levels of *Calca* (fold change = 1.76, *p* = 0.0001) and *Tac1* (fold change = 2.12, *p* = 0.0002) was observed in neurons following radiation exposure compared to the non-irradiated control (Figure 5a,b). Similarly, osteoclast activity markers *Ctsk* (fold change = 2.07, *p* = 0.0264) and *Mmp9* (fold change = 2.04, *p* = 0.0127) were significantly upregulated in osteoclasts after radiation exposure in the co-culture model (Figure 5c,d). Additionally, when co-cultures were treated with risedronate, a marked inhibition of radiation-induced osteoclast activity was evident, as indicated by decreased expression of *Ctsk* and *Mmp9* (Figure 5c,d). Interestingly, the presence of risedronate in co-cultures also mitigated the radiation-induced increase in expression of *Calca* (~30% reduction, *p* = 0.165) and *Tac1* (~40% reduction, *p* = 0.088) (Figure 5e,f). These findings collectively suggest that bisphosphonate intervention can effectively counteract the radiation-induced increase in pain-associated neuropeptides, presumably by inhibiting osteoclast activity. These findings further suggest that the reduction in neuropeptide expression by risedronate in co-cultures post-radiation exposure predominantly arises from its inhibitory effect on osteoclasts.

### 3.5. Effect of Direct Radiation Exposure on Sensory Neurons and Neuropeptide Expression

Previous investigations primarily focused on elucidating the impact of activated osteoclasts on neuronal cultures. However, considering the clinical context where both ribs and nerves are concurrently exposed to radiation near targeted tumors, it becomes imperative to understand the direct effects of radiation on neurons and its potential role in chest wall pain post-radiation.

**Figure 6:**
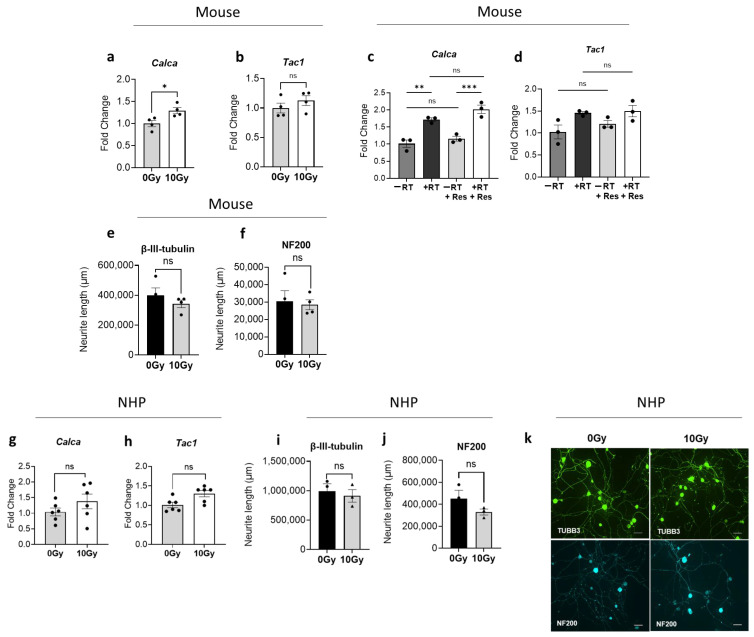
Direct effects of radiation on neuropeptide expression in sensory neurons. (**a**,**b**) mRNA expression levels of CGRP/*Calca* and SP/*Tac1* in murine sensory neuronal cultures directly exposed to 10 Gy Cs-137 γ-radiation compared to unexposed cells (0 Gy). The experiment was performed two times with four biological replicates. (**c**,**d**) Gene expression levels of CGRP/*Calca* and SP/*Tac1* in sensory neurons following radiation and pre-treatment with risedronate (50 µM). The experiment was performed two times with two biological replicates. The bar graph displays representative results from one independent experiment. (**e**,**f**) Analysis of neurite length post-radiation in murine sensory neuronal cultures stained with antibodies against β-III tubulin and NF200 by immunofluorescence. The experiment was conducted two times in quadruplicate. Unpaired Student’s *t*-test was used for statistical analysis. (**g**,**h**) mRNA expression levels of CGRP/*Calca* and SP/*Tac1* in non-human primate sensory neuronal cultures directly exposed to 10 Gy Cs-137 γ-radiation compared to unexposed cells (0 Gy). The experiment was performed once with six biological replicates. The bar graph displays representative results from one independent experiment. (**i**,**j**) Neurite length analysis post-radiation in NHP sensory neuronal cultures. (**k**) Representative immunofluorescence images of NHP sensory neurons stained with neuronal markers, β-III tubulin (TUBB3), and NF200. Scale bar = 100 μm. Unpaired Student’s *t*-test was used for all statistical analyses except for (**c**,**d**), which were analyzed using one-way ANOVA with Tukey’s post hoc. Levels of statistical significance are indicated as * *p* ≤ 0.05, ** *p* ≤ 0.005, and *** *p* ≤ 0.0005. ns: not significant.

To address these questions, sensory neuronal cultures were exposed to 10 Gy radiation, and subsequent characterization was conducted to assess nerve growth and gene expression changes. Analysis performed 48 h post-radiation through RT-qPCR revealed that direct radiation exposure led to an increase in the mRNA expression of neuropeptides *Calca* and *Tac1*, with particularly notable elevation in *Calca* levels reaching statistical significance (fold change: 1.29, *p* = 0.0244) (Figure 6a,b). As expected, risedronate pre-treatment exhibited no discernible effect on neuropeptide expression (Figure 6c,d). In addition, immunofluorescence studies indicated that the change in total neurite length of sensory neurons stained with β-III tubulin and NF200 post-radiation was not statistically significant (Figure 6e,f). Similar observations were made in neuronal cultures derived from two different non-human primates exposed to the same radiation dose, though none of the changes reached statistical significance (Figure 6g–k).

### 3.6. Impact of Osteoclast-Derived Secretory Factors on Irradiated Neurons

**Figure 7:**
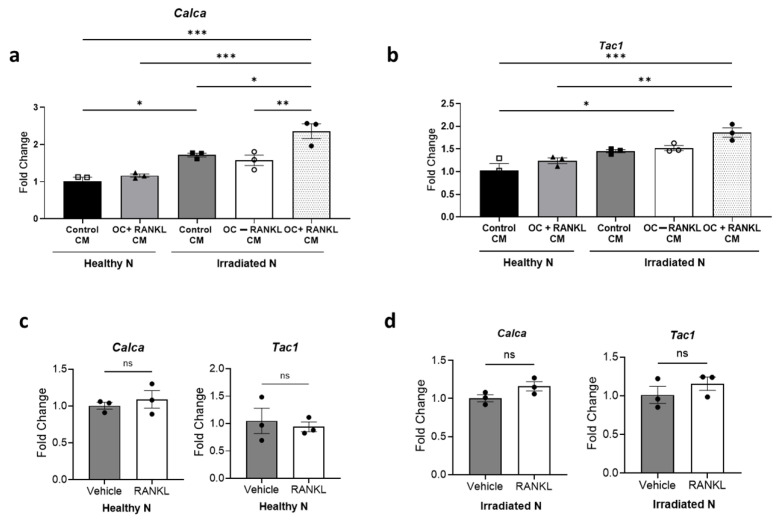
Enhanced neuropeptide expression by osteoclast-derived factors in irradiated murine neurons. (**a**,**b**) Expression levels of CGRP/*Calca* and SP/*Tac1* in murine DRG sensory neurons, either non-irradiated or irradiated murine sensory neurons, either non-irradiated or irradiated with 10 Gy Cs-137 γ-radiation, and subsequently exposed to C.M from control or RANKL-differentiated osteoclasts (induced by 35 ng/mL RANKL). One-way ANOVA with Tukey’s post hoc test was used for statistical analysis. (**c**,**d**) Impact of direct RANKL treatment (35 ng/mL) on neuropeptide mRNA expression in healthy non-irradiated and irradiated sensory neurons. Unpaired Student’s *t*-test was used for statistical analysis. All experiments were performed two times with three biological replicates. The bar graph displays representative results from one independent experiment. Levels of statistical significance are indicated as * *p* ≤ 0.05, ** *p* ≤ 0.005, and *** *p* ≤ 0.0005. ns: not significant.

Recognizing that damaged neurons are more sensitive to surrounding factors due to altered signaling pathways, increased excitability, and impaired repair mechanisms, we hypothesized that irradiated neurons may respond differently to factors secreted by osteoclasts than non-irradiated neurons. Understanding the complexity of interactions between these two cell types in the clinical setting after SBRT, where tissues near the treatment site may interact with those farther away, prompted us to explore the impact of secretory factors from adjacent bone cells on damaged nerves. Irradiated and non-irradiated sensory neuronal cultures were exposed to C.M from mature osteoclasts (non-irradiated). We observed that the C.M derived from RANKL-induced mature osteoclasts did not affect the expression level of both *Calca* and *Tac1* in non-irradiated (healthy) sensory neurons. In contrast, sensory neurons that were irradiated prior to exposure to the same osteoclast-derived C.M showed higher expression of both *Calca* (fold change = 1.37, *p* = 0.026) and *Tac1* (fold change = 1.28, *p* = 0.064) compared to those exposed to control C.M (Figure 7a,b). To ascertain whether the increase in peptide expression induced by RANKL-induced osteoclast C.M was solely attributed to RANKL, we treated both healthy and irradiated neurons with RANKL alone. However, no change in peptide expression was observed (Figure 7c,d).

## 4. Discussion

Our study explores the relationship between osteoclasts and sensory neurons after exposure to high dose radiation (10 Gy), and we aimed to identify potential mechanisms that underlie the development of CWP following SBRT to tumors near the chest wall. First, we demonstrated that high dose radiation stimulated osteoclast maturation and activity in agreement with our prior low dose per fraction rodent models [24,35]. The resulting dysregulation in bone turnover compromises structural integrity and likely is the primary cause of SBRT-induced rib and vertebral fractures.

While RIRF manifests as acute, severe CWP that often takes months to resolve, patients commonly report both acute and chronic CWP even in the absence of any radiologically apparent fractures [4,36]. Potential mechanisms of post-SBRT CWP without clear fracture include acute localized inflammation, occult microfractures, fibrosis of subcutaneous soft tissues, and/or direct injury to intercostal nerves.

Routine therapeutic interventions include escalating trials of anti-inflammatory medications (non-steroidal anti-inflammatory medications, or corticosteroids), and narcotics [14,37,38]. Usually, these are effective strategies, and pain is self-limited over weeks to months, but treatment may lead to unwanted side effects including gastric ulcers, renal/liver toxicity, immunosuppression, and, in the case of opioids, opioid use disorder [39]. In the setting of chronic refractory post SBRT pain, intercostal nerve block or gabapentin have been utilized, with the efficacy of the latter suggesting a neuropathic mechanism [8,40]. Welsh et al. [8] described a correlation between pre-SBRT obesity and diabetes with higher rates of chronic CWP development, suggesting that underlying subclinical or clinical neuropathy may lower the threshold for radiation-induced nerve damage to contribute to CWP.

Prior studies outside of the context of bone irradiation have validated that upregulated osteoclast activity from other pathologies such as osteoporosis, osteoarthritis, and lower back pain can modulate the expression of CGRP, SP, and/or NGF, contributing to pain sensitivity [41,42,43,44]. Additionally, increased abnormal nerve sprouting has been linked to osteoclast activity [28,45]. Together, these findings suggest that osteoclasts contribute to pain in more complex ways than just loss of bone structural integrity through resorption.

**Figure 8:**
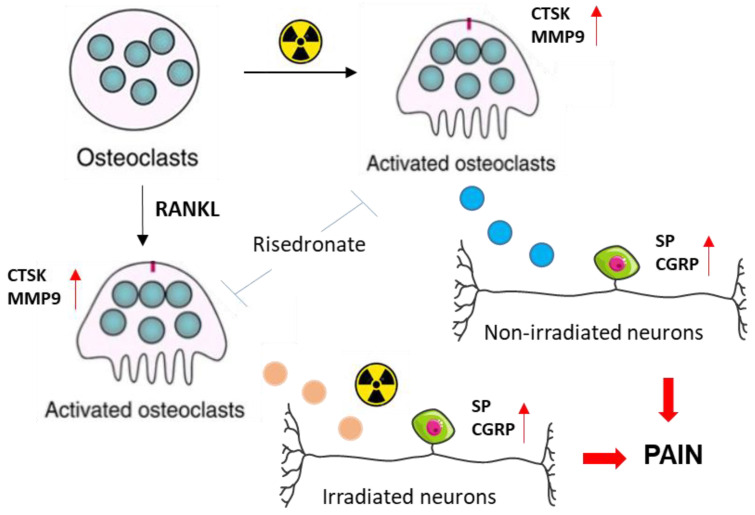
Schematic overview of osteoclast and sensory neuron interaction in radiation-induced chest wall pain. High-dose radiation treatment increases the activity of mature osteoclasts, as indicated by increased expression of osteoclast biomarkers including *Ctsk* and *Mmp9*. C.M derived from radiation-activated osteoclasts increases the expression of pain-associated neuropeptides (CGRP/*Calca*, SP/*Tac1*) in non-irradiated sensory neurons. Sensory neurons exposed to high-dose radiation also exhibit increased expression of CGRP/*Calca* and SP/*Tac1* when exposed to secretory factors released from RANKL-activated osteoclast cells. The increased expression of pain-associated biomarkers in neurons is reduced when osteoclast activity is inhibited by risedronate.

The present study is unique in demonstrating substantial crosstalk between radiation-activated osteoclasts and sensory neurons, both irradiated and unirradiated. Importantly, we delivered 10 Gy, as this dose was previously shown to cause rapid bone loss in NHPs for a study delivering SBRT-relevant thoracic irradiation [46]. We demonstrated that irradiation enhanced osteoclast maturation and activity, as indicated by increased expression of markers such as *Ctsk* and *Mmp9*. C.M from these activated osteoclasts significantly increased the expression of *Calca* and *Tac1* in sensory neurons, genes that encode the neuropeptides CGRP and Substance P, which are known to promote pain via neurogenic inflammation, peripheral sensitization, and enhanced nociceptor excitability [47,48]. This effect was attenuated by bisphosphonate treatment. In addition, irradiated neurons exhibited greater induction of *Calca* and *Tac1* when exposed to C.M from RANKL-activated osteoclasts, suggesting that prior radiation exposure sensitizes neurons to osteoclast-derived factors. Together, these findings support a model in which osteoclasts modulate pain signaling at the bone–nerve interface. This mechanistic pathway is summarized in Figure 8 and proposes that osteoclast activation contributes to radiation-induced CWP through both inflammatory and neuropathic mechanisms.

While the mechanism by which osteoclast-derived factors increased neuropeptide levels is not clear, several signaling pathways may be involved, including p38 MAPK and NF-kB [49,50,51]. In addition to upregulation of neuropeptides, additional molecular changes contributing to functional sensitization are likely. There is evidence from animal studies that acute radiation exposure can increase levels of pain-relevant ion channels, such as TRPV1 in sensory neurons, particularly in mucosal tissues [52,53]. Future mechanistic studies could explore signaling pathways and alterations in pain-relevant ion channels, which represent an interesting and promising area for further investigation.

Interestingly, in our study, we did not observe change in neurite length in sensory neurons when mice or NHP-derived DRG neurons were exposed to C.M from irradiated osteoclasts. These results suggest that activated osteoclasts do not release trophic factors that induce sprouting or actual structural changes in sensory neurons. Alternatively, our in vitro system, which lacks supporting cells (e.g., glial, immune cells), may have limited our ability to detect morphological changes in neurite structure as these cell types have been shown to contribute to neuronal remodeling in vivo [54,55]. These findings highlight the complexity of sensory neuron responses to radiation and osteoclast-derived factors and warrant further investigation.

Our prior clinical data seems to support this osteoclast neuron cross talk mechanism as well. In a randomized double-blind clinical trial at our institution, 76 patients receiving SBRT for peripheral lung tumors were treated with placebo or risedronate (150 mg single dose 2 weeks prior to SBRT). While there was no difference in the rate of RIRF (overall 34%), risedronate was associated with a significant reduction in pain, which is recognized as adverse even in the Common Terminology Criteria for Adverse Events (CTCAE), with fewer patients experiencing grade 2 or higher CWP (42 vs. 18%, *p* = 0.045) [56] (Published abstract). As the first study to evaluate the use of bisphosphonates to reduce the risk of radiation-induced bone loss after peripheral lung SBRT, a standard dosing regimen for the bone-protecting agent was not available at the time of trial design, and it is possible that the dose of risedronate was inadequate, which may explain the similar RIRF rates. If radiation-mediated activation of osteoclasts does in fact contribute to both RIRF and at least some components of CWP without fractures, this presents osteoclast inhibition as an attractive potential therapeutic target for patients with tumors adjacent to bone and nerve.

Our current study findings emphasize the therapeutic potential of osteoclast inhibitors using bisphosphonates in managing CWP. Risedronate, which inhibits osteoclast activity, significantly decreased the expression of neuropeptides induced by osteoclast-derived factors in neurons. Although risedronate did not directly affect the *Calca* and *Tac1* levels, they increased after radiation exposure in neurons, and it significantly inhibited *Calca* and *Tac1* in sensory neurons in co-culture with osteoclasts after radiation treatment. The extent to which direct radiation exposure and the neuropeptide and nerve growth changes contribute to CWP is unclear and warrants further study. The neuropeptide increase induced by osteoclast-derived factors and/or direct irradiation might lead to different functional outcomes. It would be important to better understand the component of CWP caused by direct nerve damage after SBRT versus an alternative pathway in which neuropeptides released from neurons near activated osteoclasts act on pain signaling.

While this study focused on establishing mechanistic plausibility using in vitro systems, we recognize the importance of validating these findings in vivo. Animal models are essential for evaluating behavioral correlations of CWP, dose-dependence, and potential off-target effects of osteoclast inhibition. To that end, we are currently pursuing animal models of high-dose exposure to the chest wall concurrent with various doses/schedules of bisphosphonates to identify a prophylactic effect on CWP or RIRF and to elucidate an appropriate agent and dose for future clinical study. A better understanding of the agent, dose, and timing most suitable for future study, would inform the design of a larger trial specifically powered for reduction in CWP/RIRF for patients with tumors immediately adjacent to the chest wall planned for SBRT. If successful, this would be a valuable and immediately implementable strategy for clinicians to reduce the risk of chest wall toxicity after SBRT while maintaining excellent cancer control rates, particularly given the well- established use and safety profile of bisphosphonate therapy in other diseases.

Our study has several limitations. First, the in vitro nature of our experimental model, though valuable for identifying cell-specific mechanisms, does not fully capture the complexity of dynamic bone–nerve interactions that occur in vivo. Additionally, several in vitro experiments were performed less than 3–4 times due to the exploratory nature of the study and resource constraints. Nevertheless, these preliminary findings are supported by clinical trial results [56], which suggest similar underlying biological mechanisms. Second, while the inclusion of NHPs was intended to enhance translational relevance, the available NHP tissue cohort was limited in sample size and heterogeneous with respect to prior radiation exposure. These factors reduce statistical power and generalizability, though internal validity was preserved by directly comparing the irradiated NHP to a concurrently studied control. Third, our study did not include functional assessments of pain, such as calcium imaging or electrophysiological recordings, which limits our ability to directly link molecular changes to neuronal excitability or pain perception. However, our findings serve as a mechanistic bridge between clinical observations and future preclinical validation. To address these limitations, ongoing and planned studies will involve expanded in vitro replication with additional intervention arms, in vivo rodent models to evaluate bone-nerve interaction outcomes, and larger, more homogeneous NHP cohorts with integrated pain assays.

## 5. Conclusions

Our study presents unique evidence that radiation-induced osteoclast activation results in crosstalk with neurons that may contribute to CWP after lung SBRT near the chest wall, particularly in patients without evidence of fracture. These findings suggest that osteoclast inhibition may represent a novel strategy not only to reduce radiation-induced bone loss and fracture but potentially prevent or relieve CWP through neuromodulation of osteoclast-derived, radiation-induced pain signaling. In addition, while our study focuses on CWP following SBRT to peripheral lung tumors, the mechanisms we describe may be broadly relevant to other clinical scenarios involving high-dose radiation near bone–nerve interfaces, such as pelvic or vertebral irradiation. Like the chest wall, pelvic and vertebral regions also contain dense innervation adjacent to bone and are susceptible to radiation-induced toxicity. Osteoclast activation and nociceptive nerve fiber involvement have been implicated in pain syndromes in these regions, including sacral plexopathy and vertebral osteoradionecrosis, suggesting potential mechanistic overlap that warrants targeted investigation. To translate these findings clinically, future research should prioritize in vivo validation of osteoclast–neuron interactions in radiation models and test optimized bisphosphonate dosing regimens for CWP prevention. Additional studies are also needed to define timing, duration, and long-term safety of osteoclast-targeted interventions, particularly in vulnerable populations such as elderly patients. These efforts may guide rational design of clinical trials and ultimately improve supportive care for patients undergoing SBRT near critical bone-nerve interfaces.

## Data Availability

The datasets used and/or analyzed during the current study are available from the corresponding author on request.
